# A Phase I study to evaluate the safety and antitumor activity of durvalumab (MEDI4736) in combination with tremelimumab in patients with advanced solid tumors

**DOI:** 10.1186/2051-1426-3-S2-P165

**Published:** 2015-11-04

**Authors:** Kyriakos P Papadopoulos, Frank Tsai, Omid Hamid, Feng Xiao, Keith E Steele, Marlon C Rebelatto, Paul B Robbins, Joyson J Karakunnel, Dominic W Lai, Amit Mahipal

**Affiliations:** 1 South Texas Accelerated Research Therapeutics (START), San Antonio, TX, USA; 2 Pinnacle Oncology Hematology, Scottsdale, AZ, USA; 3 The Angeles Clinic and Research Institute, Los Angeles, CA, USA; 4 MedImmune, Gaithersburg, MD, USA; 5 Moffitt Cancer Center, Tampa, FL, USA

## Background

The immune system is able to control the growth of many types of cancers. Most tumors show infiltration by tumor infiltrating lymphocytes, but tumors modulate the local microenvironment by expressing immune-inhibitory molecules. Blockade of immune checkpoints such as cytotoxic T-lymphocyte-associated antigen-4 (CTLA-4), programmed cell death-1 (PD-1), and programmed cell death ligand-1 (PD-L1) has shown clinical activity in immune-responsive tumors as well as those historically deemed unresponsive to treatment. Durvalumab (MEDI4736) is a selective, high affinity human IgG1 monoclonal antibody (mAb) that blocks PD-L1 binding to PD-1 (IC_50_ 0.1 nM) and CD80 (IC_50_ 0.04 nM). Tremelimumab is a selective human IgG2 mAb inhibitor of CTLA-4 which promotes T-cell activity through CTLA-4 inhibition. Both durvalumab and tremelimumab have demonstrated acceptable safety profiles and promising clinical activity as single agents in multiple tumor types. Recent data suggest that combined checkpoint inhibition may generate superior antitumor activity versus monotherapy, resulting in higher, deeper, and more durable response rates, which suggests that dual inhibition may overcome barriers to immune response in historically non-immune responsive tumors. We describe an ongoing study assessing the safety and antitumor activity of durvalumab in combination with tremelimumab in patients with advanced solid tumors.

## Methods

This is a Phase I, multicenter, open-label, dose-exploration and dose-expansion study in patients with select advanced solid tumors (NCT02261220). Eligible patients (≥18 years) will have recurrent or metastatic (R/M) disease and may have been previously treated in the R/M setting. The primary objective of this study is to assess the safety and tolerability, and maximum tolerated dose of durvalumab in combination with tremelimumab. The secondary objectives are to assess antitumor activity (including overall response rate, disease control rate, duration of response, progression-free survival, and overall survival), pharmacokinetics, pharmacodynamics, and immunogenicity of the combination. Exploratory objectives will include an assessment of biomarkers that may correlate with clinical activity of durvalumab in combination with tremelimumab. Recruitment is ongoing, with an estimated enrollment of 225–240 patients.

## Trial registration

ClinicalTrials.gov identifier NCT02261220.

